# Data for electricity consumption, thermo-physical characteristics of residential buildings in Tehran

**DOI:** 10.1016/j.dib.2022.107813

**Published:** 2022-01-08

**Authors:** Mahsa Torabi, Sahar Labbafan, Bahareh Farajnia

**Affiliations:** a Faculty of Architecture, University of Tehran, Tehran, Iran; b Faculty of Architecture, Pars University, Tehran, Iran

**Keywords:** Monthly electricity consumption, Building physical characteristics, Residential buildings, Real-time data

## Abstract

This data article presents data related to 100 residential units in Tehran, Iran. For each building the ``average monthly electricity consumption'' data for 190 months has been provided. In addition, some physical characteristics of each building are given in the dataset. Presented data includes collected electricity end-users in residential units as well as thermos-physical characteristics of the buildings through a structured questionnaire. The physical characteristics covered in this study include; the number of floors, morphology of the yard and the ratio of the windows on each side window material, area, floor number of the studied residential unit, location in the city, cooling system, heating system, and so on. Information provided in this data article can be useful for research on energy prediction studies and also energy management strategies, and policy making to achieve sustainability targets. Having few datasets published on this topic, especially in hot and arid geographic regions, authors believe that the result of this data study can be generalized to the larger region of the Middle East and North Africa (MENA). The data consists of two parts; 1. Physical specifications of the building and 2. History of electricity consumption.

## Specifications Table

**Table:** 

Subject	Engineering
Specific subject area	Electricity consumption in buildings
Type of data	Table
How data were acquired	Data related to monthly electricity consumption has been obtained through the Tehran Electricity Department record^1^; The data related to the physical characteristics of the building was obtained through a questionnaire filled out by the residents of the same residential units.
Data format	Raw, filtered
Description of data collection	Data were obtained through a questionnaire. The questionnaire was filled out by the residents of the residential buildings; and the amount of monthly energy consumption has been obtained through the electricity ID of the residential unit (which is the code specific to that residential unit and has been registered by the person who filled in the questionnaire) and the website of the Tehran Electricity Authority. In total, 14 attributes are defined for each residential unit, which usually have the greatest impact on electricity consumption among all the features of the building; These items are related to the overall shape and size of the building and the residential unit, window specifications, yard morphology, cooling and heating systems and building location. The number of months with average monthly consumption in the dataset for each residential unit depends on the year in which the building was built and put into operation; It is noteworthy to mention that the electricity consumption has been recorded on the website and system of Tehran Electricity Company.
Data source location	Tehran, Iran
Data accessibility	With the article

1 (tbtb.ir).

## Value of the Data

• Real-time data can be used to build a machine learning model to predict the energy consumption of residential buildings in Tehran or another city in the world that has a climate and urban texture similar to Tehran. (Regardless of user behavior).
• The real-time data presented in this article are useful in the development of energy management strategies and for the design, measurement, modeling and simulation of energy systems in buildings to be constructed in the future or to be replaced in existing buildings.
• To use data in future research, four factors must be considered, including the date of power consumption, the amount of power consumption, the physical characteristics of the building, and the location.
• The real-time data presented in this article is useful for architects, designers and electrical engineers.
• The real-time data presented in this article can be used as an independent variable to model the electricity consumption of residential units in Tehran.

## 1 Data Description

**Fig. 1.**
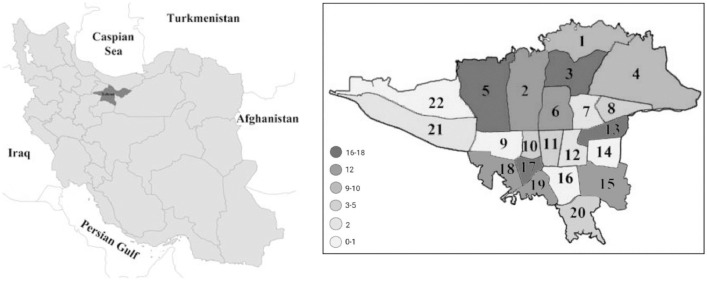
Frequency of samples among 22 districts of Tehran.

The data provided is collected from the metropolis of Tehran, Iran. Fig. 1 shows the location of Tehran with its comprising 22 municipal districts. In the picture below, the distribution of buildings through different districts can be seen. Districts with high populations, have more samples and are illustrated in a darker color. Data from sample buildings were collected using a questionnaire that is provided as a supplementary file.

There are two sheets in the data set file; The first sheet contains the following variables:

• number_of_floors: Number of floors of the residential building.
• your_municipal_district: A district of Tehran where the building is located.
• building_yard_morphology: Type of yard placement relative to the external walls of the building.
• electricity_bill_id: The ID through which the power consumption is extracted from the website.
• construction_date: Year of construction of the building.
• unit_floor_number: The floor on which the residential unit is located.
• unit_area: The area of the desired residential unit.
• windows_morphology: Type of window placement in the external walls. of the building.
• N_WWR_in_unit: Percentage of openings on the north front of the building.
• E_WWR_in_unit: Percentage of openings on the east front of the building.
• S_WWR_in_unit: Percentage of openings on the south front of the building.
• W_WWR_in_unit: Percentage of openings on the west front of the building.
• windows_material: Window frame material and window glass type.
• cooling_system: The type of cooling system.
• heating_system: The type of heating system.

There are two variables in the second sheet:

• Time_Stamps: Different months of each year when the building unit has electricity consumption and its consumption is recorded on the website.
• Mean_Monthly_Consumption: Average monthly electricity consumption.

**Fig. 2:**
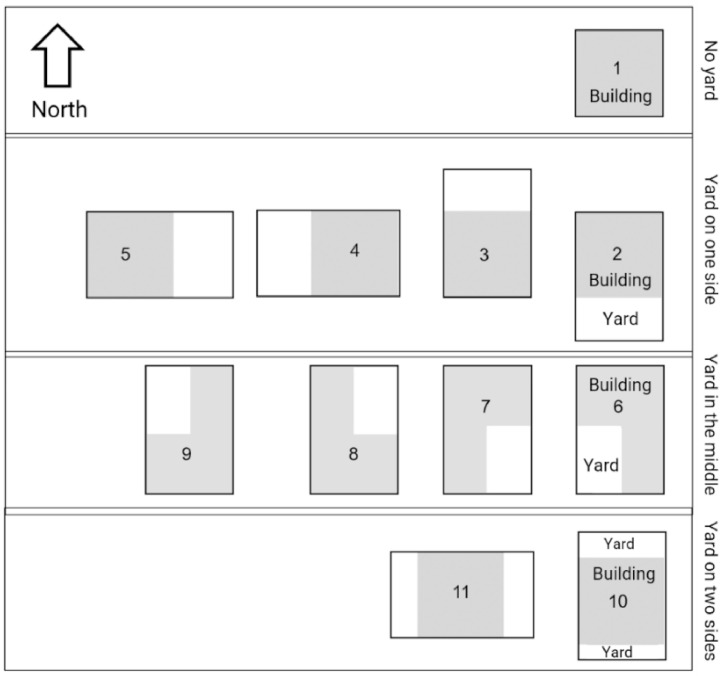
Yard morphology.

One of the questions of the questionnaire was the morphology of the yard. As Fig. 2 shows each item shows how the building is positioned in relation to the yard. Therefore, the nearest picture to the building morphology was selected by the participant and the number was filled in the questionnaire.

**Fig. 3:**
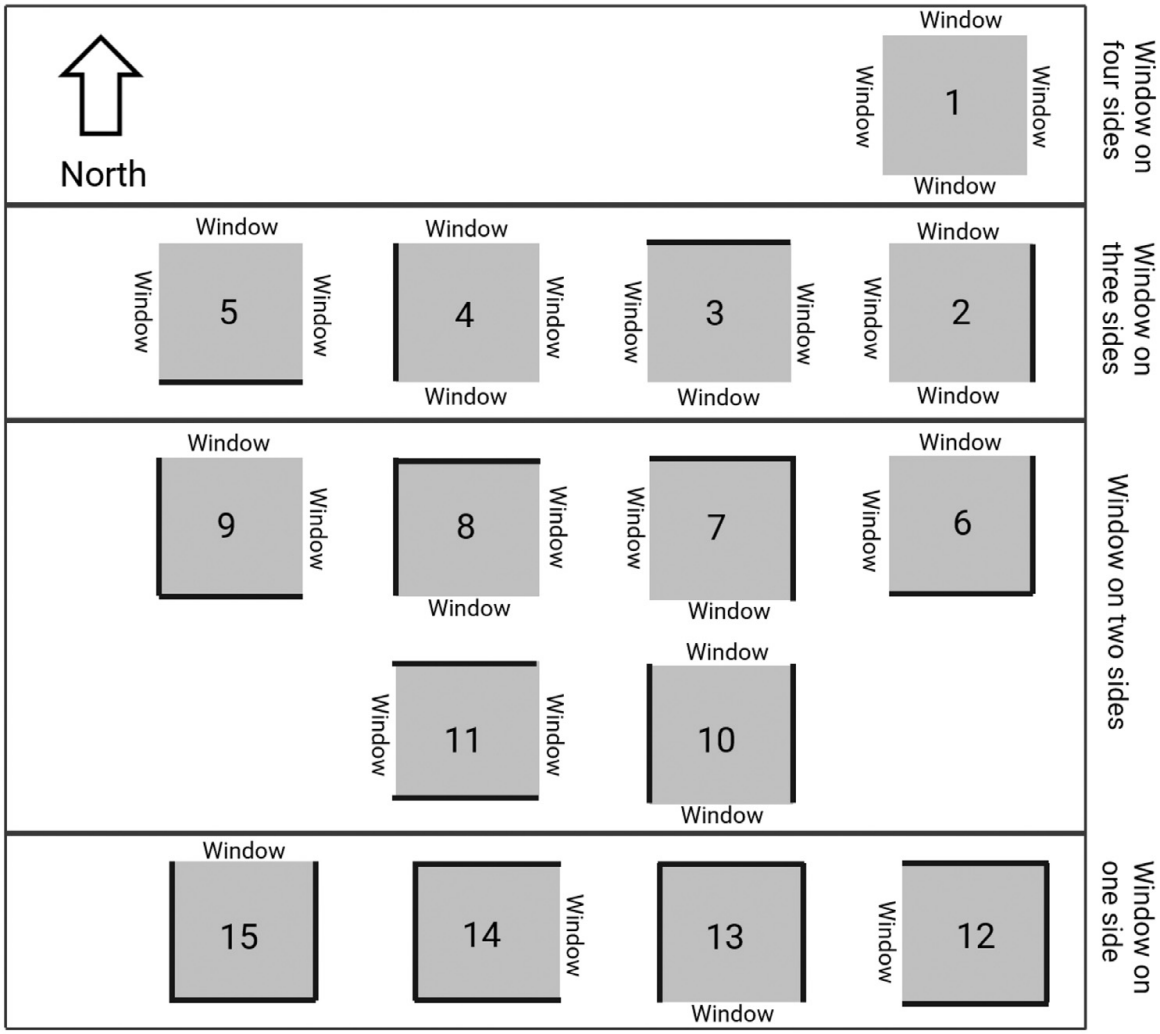
Windows morphology.

**Chart 1:**
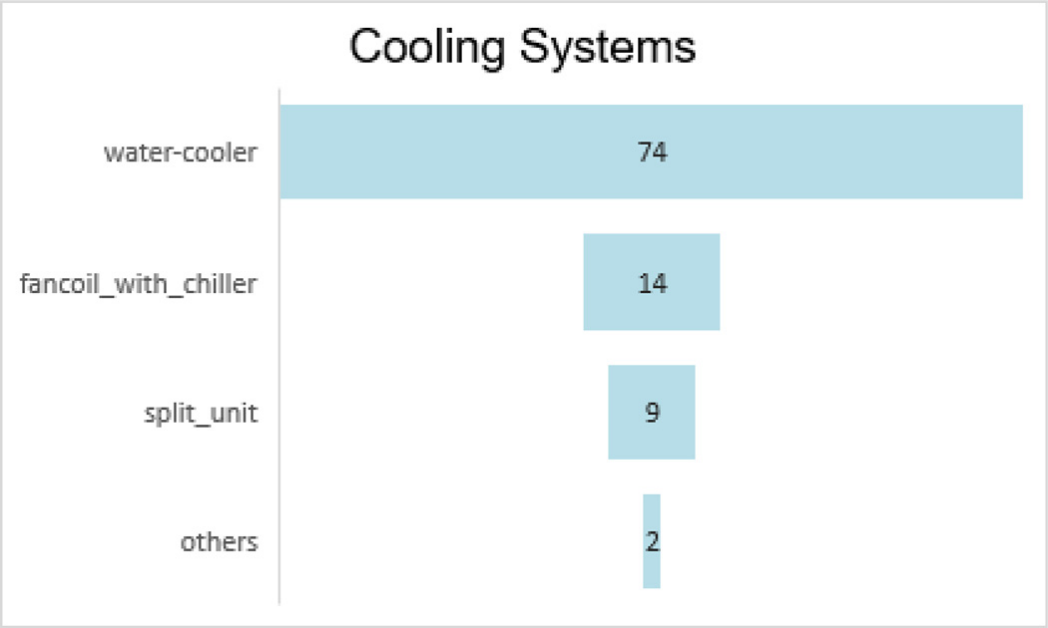
The frequency of different cooling systems.

**Chart 2:**
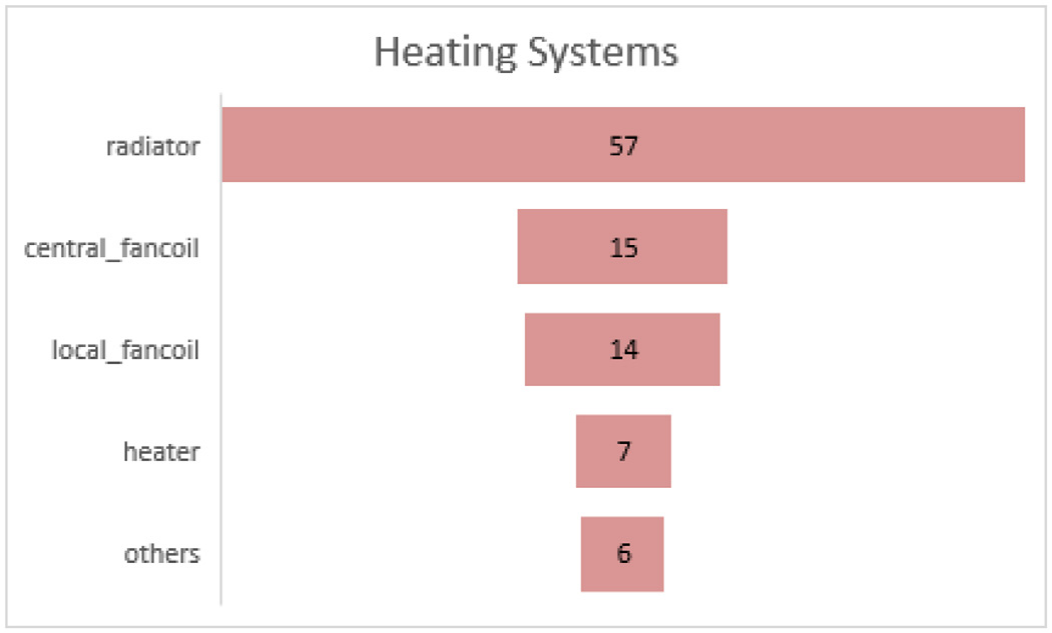
The frequency of different heating systems.

Another question was window morphology. Similarly, Fig. 3 shows each item demonstrating a conventional morphologic pattern. The participant could choose the item which best describes their case and use the number as the answer to the question. This is an error-free way for all walks of life to report the physical characteristics of their buildings in the dataset. Moreover, Chart 1 and Chart 2 demonstrate the frequency of the most common heating and cooling equipment for sample buildings of the Tehran metropolis.

**Chart 3:**
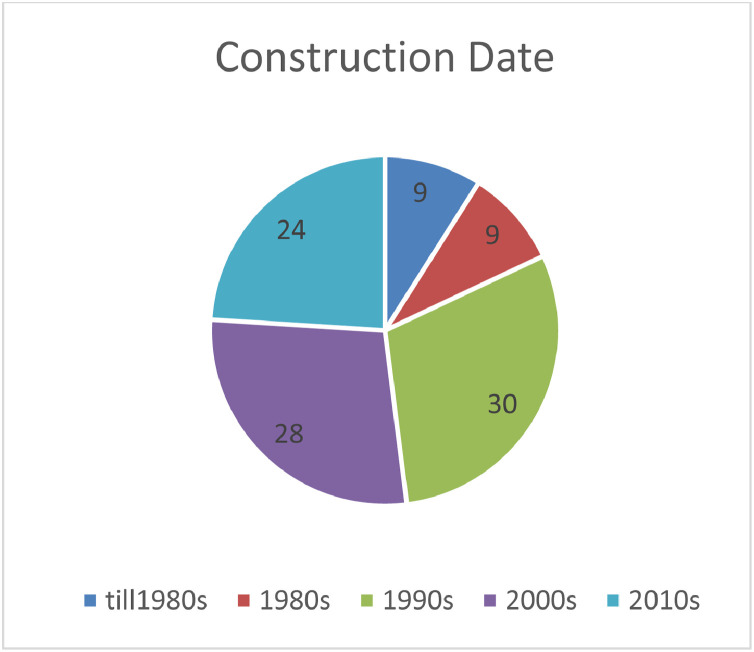
The frequency of “year of construction of residential units” in the samples.

As mentioned previously, the number of months with average monthly consumption depends on the length of operation stage of the building which is directly related to the ``year of construction of the building''. In other words, the older the building, the longer electricity consumption record is available. Chart 3. Shows the frequency of buildings in the dataset according to their construction year.

**Chart 4:**
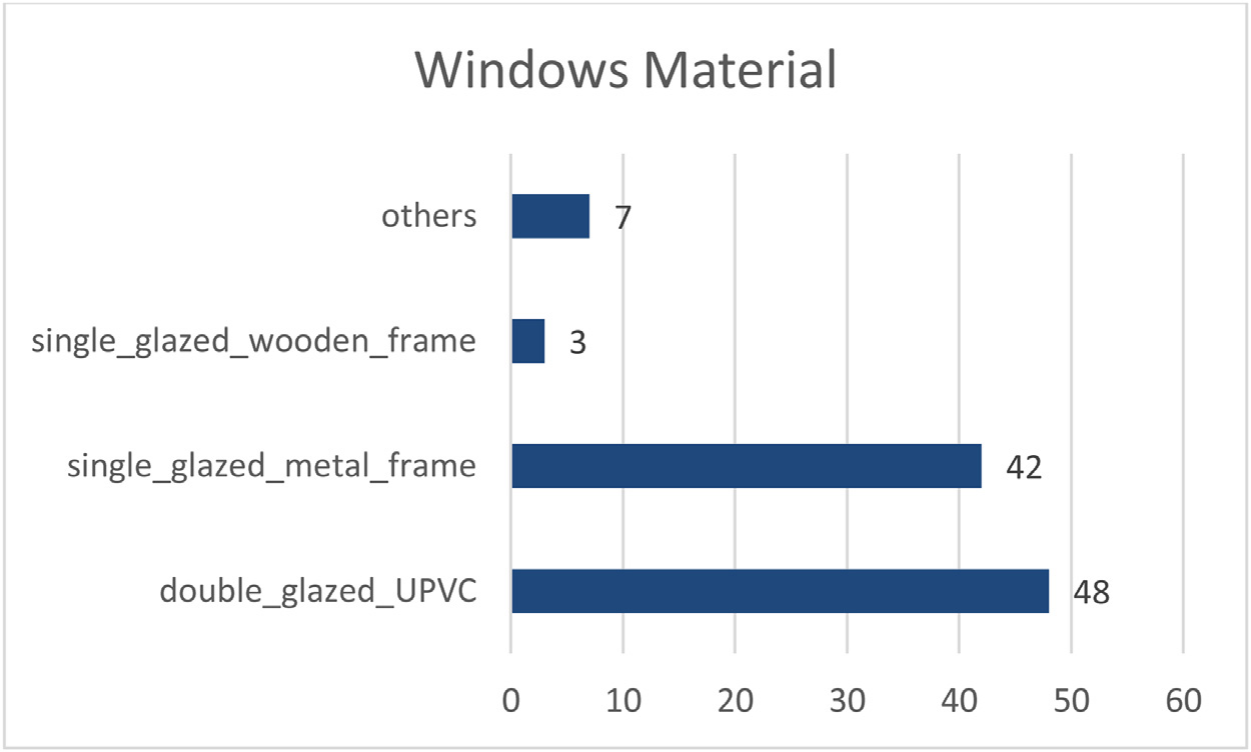
Comparison between the frequency of “different window materials” in the samples.

Applicants were asked to fill in the questionnaire and choose the window material of their residential unit. There are four options to choose from according to common window material in Tehran: Double glazed UPVC, Single glazed material frame, Single glazed wooden frame and others. Chart 4. Shows the frequency of used windows in the database.

**Chart 5:**
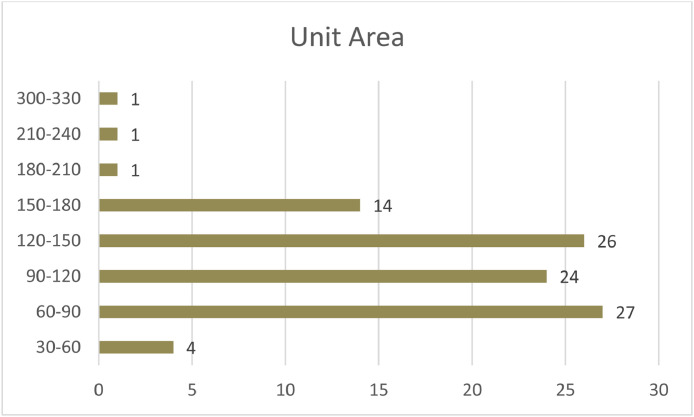
Comparison between the frequency of “Area of residential units” in the samples.

Another factor, which has a direct impact on electricity consumption, is building area. In this research, a numerical continuous range was defined primarily. According to the conventional residential units of Tehran, the range of 30–330 with steps of 30 m^2^ was defined and the participant could allocate their unit to the appropriate class accordingly. Chart 5. Shows the distribution of sample buildings according to their area.

**Chart 6:**
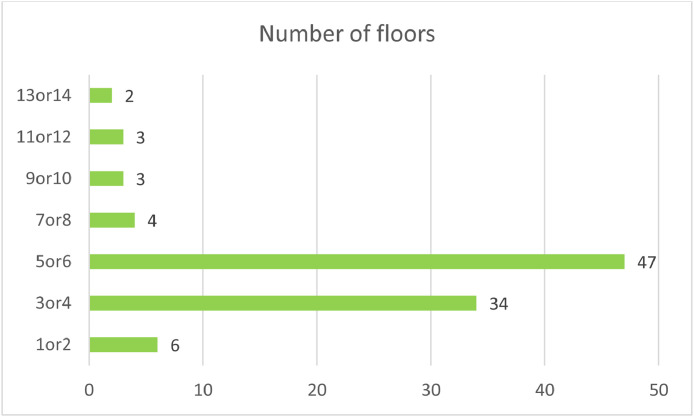
Comparison between the frequency of “different number of floors” in the samples.

Lastly, the number of floors of a building was investigated. For this parameter, a range between 1 (for single-family housing) and 14 (for highrise residential buildings) was considered according to the record of issued construction permits in Tehran. This parameter was classified in classes of 1 or 2, 3 or 4, 5 or 6, etc. Chart 6. Show the distribution of sample buildings in this dataset.

## 2 Experimental Design, Materials and Methods

Real-time electricity consumption data belonging to a large number of residential units in Tehran are presented in this article. This data has been obtained from the website of Tehran Power Distribution Company. First, the residents of the residential units, with their consent, entered their electricity bill ID in the questionnaire; Then, by using this ID, the monthly electricity consumption related to that residential unit can be accessed. The data do not belong to a specific period and it started from the date of operation of the building, where the occupants start using the residential unit until the date of data collection for this article. Therefore, the number of values related to the average monthly electricity consumption depends on whether the building is old or new and also the number of years of operation.

In addition, items related to the physical characteristics of the building are collected via a questionnaire filled by occupants. The questions include items such as the number of floors in the building, unit area, construction year, number of basements, the morphology of the yard, area and configurations of windows, number of external walls in building envelope, window materials, patio specifications (if any), length and width of the building and Neighborhood density.

## Ethics Statement

All the people who filled in the questionnaire did so with the informed consent and in order to help the research. While the information of the participants is completely anonymous.

## CRediT authorship contribution statement

**Mahsa Torabi:** Conceptualization, Methodology, Data curation, Writing – review & editing. **Sahar Labbafan:** Data curation, Writing – original draft, Visualization. **Bahareh Farajnia:** Data curation, Writing – original draft.

## Declaration of Competing Interest

The authors declare that they have no known competing financial interest or personal relationship which have, or could be perceived to have, influenced the data reported in this work.

## Appendix Supplementary materials

Image, application 1

Image, application 2

