# Supplementary material for: Data for electricity consumption, thermo-physical characteristics of residential buildings in Tehran
Source: Data Brief. 2022 Jan 8;40:107813. doi: 10.1016/j.dib.2022.107813 (PMC8761682; doi:10.1016/j.dib.2022.107813)
Supplement: Supplementary file 2 [file mmc2.docx]

1-How many floors does your building have on the ground?

- one floor
- Two floors
- three story
- four floor
- Five floors
- Six floors

Other:

2-In which of the 22 municipal districts is your building located? -------------------------

3-What is the current occupancy in your building?

- Residential only
- Residential and office
- Residential and commercial
- Residential, office and commercial

4-How many basements is there in your building have ?

- One
- Two
- Three
- There is no basement

5-Which building is closest to your building in terms of yard morphology? Please enter its number.------


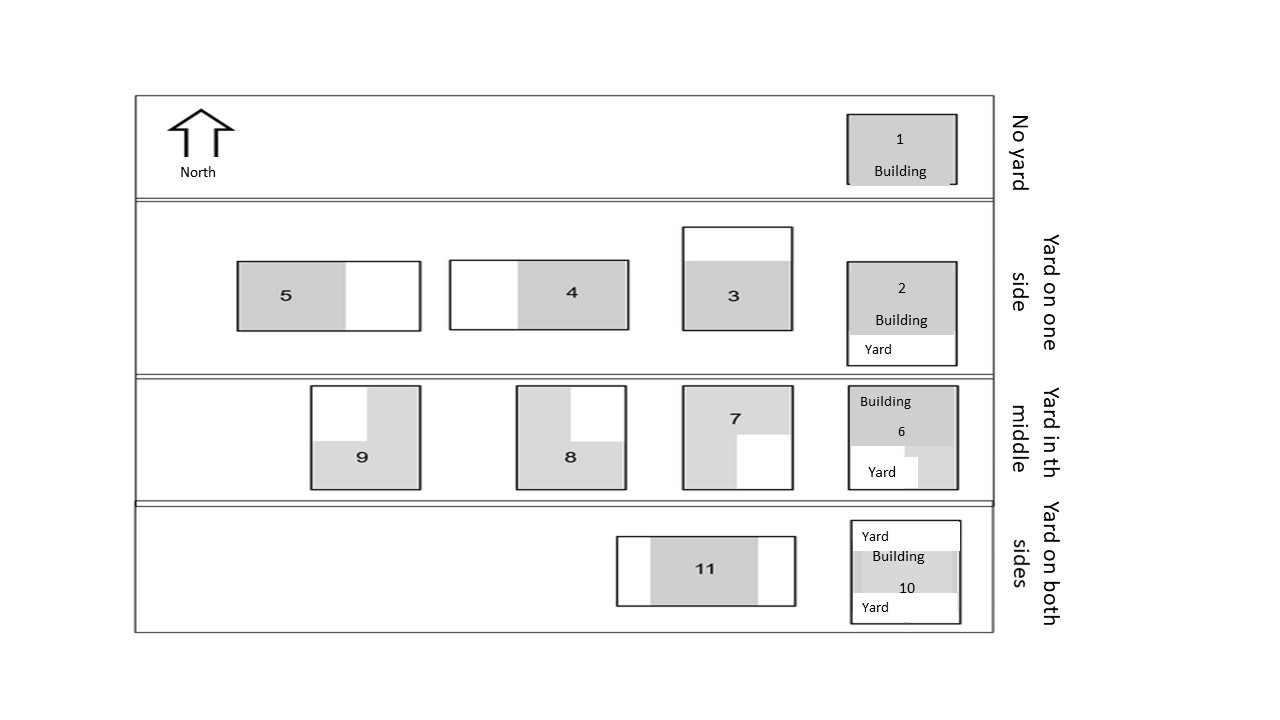


6- when was your building constructed?

Note: if you are not sure, there is a date on the electricity bill called "installation date". That show the construction year of each building

- Until the year 1360
- 60th decade
- the 70's
- 80s
- 90s

7-Enter your electricity bill ID: -------------------

Note: This information will be used to review the history of consumption.

8-Which floor is your residential unit located on?

- first floor
- second floor
- third floor
- Fourth Floor
- fifth floor
- sixth floor

9-What is the area of your residential unit?

- Less than 60 Sqm
- 60-90 Sqm
- 90-120 Sqm
- 120-150 Sqm
- 150-180 Sqm
- 180-210 Sqm
- More than 210 Sqm

10-How many faces of your unit is adjucent to the outdoors?

- One direction
- Two directions
- Three directions
- Four directions

11-Which option is closer to the window placement in your residential unit? Please enter its number.-----


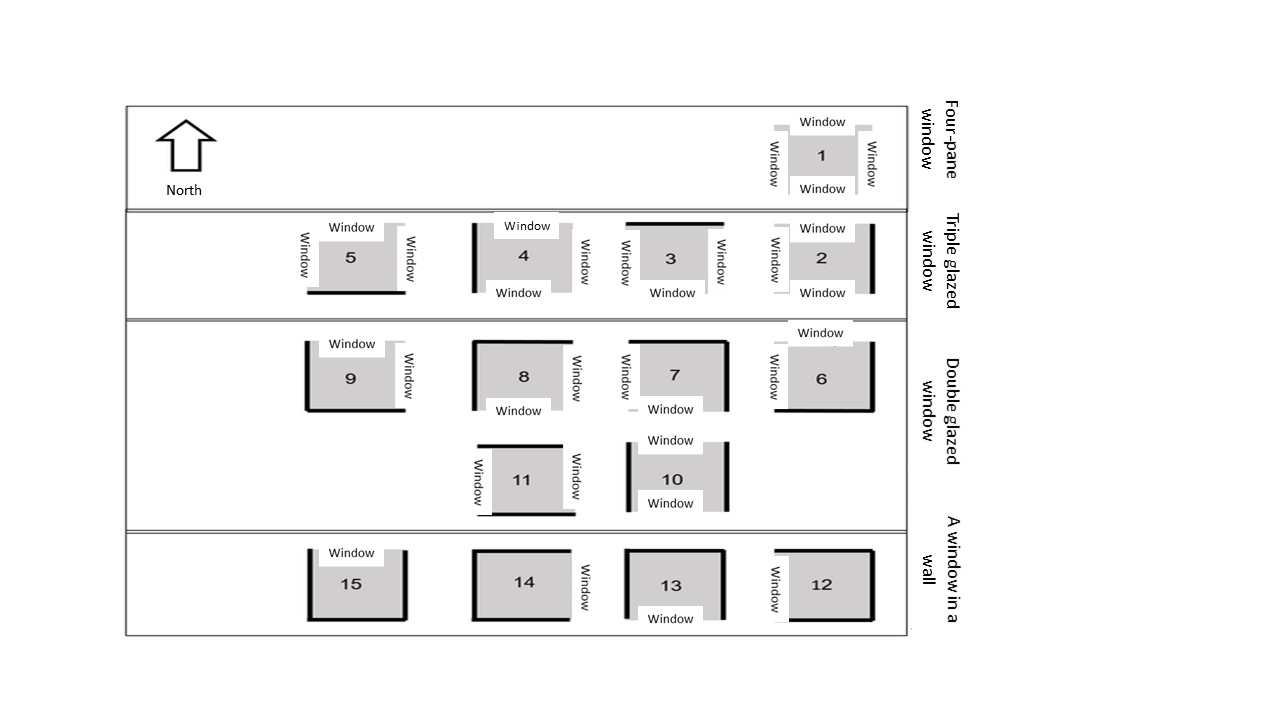


12- what percentage of the wall in each direction, is consists of window?

Note: here any transparent or operable sub surfaces is considered as a window.

*Example: 20% East 0% West 50% South 50% North*

-------- East -------- West --------South ---------North

13-What is the material of the windows in your residential unit?

- Double wall with UPVC frame
- Single wall with iron frame
- Single wall with wooden frame
- Other

14-Does your building have access to a patio or backyard?

- Yes
- No

15-If your unit is adjacent to a patio, which option is the closest to the location of the morphology of patio? Please write its number. ---------------


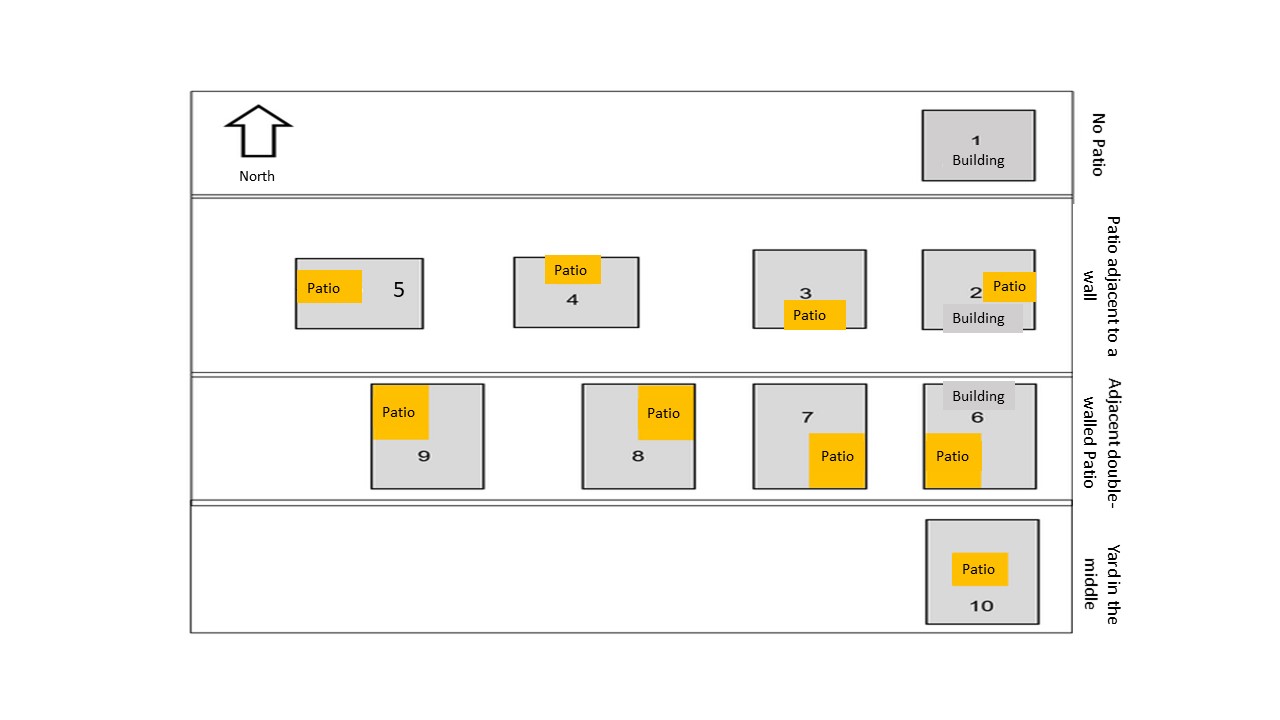


16-What is the average percentage of windows in your patio or backyard?

- Less than a quarter of a window wall.
- Between a quarter and a half is the window wall.
- Between half and three quarters of the window wall.
- More than three-quarters of the window wall.

17-What is the cooling system in your unit?

- Water Cooler
- Fan coil with chiller
- Split Unit
- Other

18-What is your unit heating system?

- Radiator
- Local fan coil
- Central fan coil
- Heater
- Other
